# Population genomic analyses reveal population structure and major hubs of invasive *Anopheles stephensi* in the Horn of Africa

**DOI:** 10.1111/mec.17136

**Published:** 2023-10-05

**Authors:** Jeanne N. Samake, Philip Lavretsky, Isuru Gunarathna, Madison Follis, Joshua I. Brown, Said Ali, Solomon Yared, Tamar E. Carter

**Affiliations:** 1 Department of Biology, Baylor University, Waco, Texas, USA; 2 Department of Biological Sciences, University of Texas at El Paso, El Paso, Texas, USA; 3 Department of Life, Earth, and Environmental Sciences, West Texas A&M University, Canyon, Texas, USA; 4 Ministry of Health Somaliland, Hargeisa, Somalia; 5 Department of Biology, Jigjiga University, Jigjiga, Ethiopia

**Keywords:** ddRAD-seq, invasive mosquito, landscape genomics, malaria

## Abstract

*Anopheles stephensi* invasion in the Horn of Africa (HoA) poses a substantial risk of increased malaria disease burden in the region. An understanding of the history of introduction(s), establishment(s) and potential *A. stephensi* sources in the HoA is needed to predict future expansions and establish where they may be effectively controlled. To this end, we take a landscape genomic approach to assess *A. stephensi* origins and spread throughout the HoA, information essential for vector control. Specifically, we assayed 2070 genome-wide single nucleotide polymorphisms across 214 samples spanning 13 populations of *A. stephensi* from Ethiopia and Somaliland collected in 2018 and 2020, respectively. Principal component and genetic ancestry analyses revealed clustering that followed an isolation-by-distance pattern, with genetic divergence among the Ethiopian samples significantly correlating with geographical distance. Additionally, genetic relatedness was observed between the northeastern and east central Ethiopian *A. stephensi* populations and the Somaliland *A. stephensi* populations. These results reveal population differentiation and genetic connectivity within HoA *A. stephensi* populations. Furthermore, based on genetic network analysis, we uncovered that Dire Dawa, the site of a spring 2022 malaria outbreak, was one of the major hubs from which sequential founder events occurred in the rest of the eastern Ethiopian region. These findings can be useful for the selection of sites for heightened control to prevent future malaria outbreaks. Finally, we did not detect significant genotype–environmental associations, potentially due to the recency of their colonization and/or other anthropogenic factors leading to the initial spread and establishment of *A. stephensi.* Our study highlights how coupling genomic data at landscape levels can shed light into even ongoing invasions.

## 1 ∣ INTRODUCTION

Malaria remains one of the leading global health concerns, with an estimated 241 million cases and over 600,000 deaths worldwide in 2020 (WHO, 2021). In Africa, members of the *Anopheles gambiae* complex have been the predominant vectors, with *Anopheles arabiensis* acting as the primary vector in parts of the Horn of Africa (HoA) which includes Djibouti, Eritrea, Ethiopia and Somalia. However, recently *Anopheles stephensi*, a natural vector in South Asian and Middle Eastern countries, is now being readily found in some East African countries and across Indian Ocean Islands (Ishtiaq et al., 2021). In Africa, *A. stephensi* was first detected in Djibouti in 2012 (Faulde et al., 2014), Ethiopia in 2016 (Carter et al., 2018), Sudan and Somaliland in 2019 (Ahmed et al., 2021; Ali et al., 2022) and Nigeria in 2020 (WHO, 2022a). Among these, the detection of *A. stephensi* in Djibouti in 2012 correlated with an exponential growth of malaria cases from 1684 in 2013 to over 60,000 in 2020 (de Santi et al., 2021; Faulde et al., 2014; Seyfarth et al., 2019; WHO, 2021). This rapid spread of *A. stephensi* led the World Health Organization (WHO) to issue a ‘vector alert’ in 2022, aiming to halt the further spread of this invasive malaria vector in Africa (WHO, 2022b).

Although often associated with urban settings, *A. stephensi* can also proliferate in rural environments due to its broad and general habitat needs during its various lifecycle stages. In particular, the ability to use any form of water storage containers, including artificial containers at the larval stage allows *A. stephensi* the ability to readily move between and within rural and urban settings (Imwong et al., 2011; Thomas et al., 2017). Along with their generalist ecology, *A. stephensi* is a highly potent malaria vector that can transmit both *Plasmodium falciparum* and *P. vivax* (Balabaskaran Nina et al., 2017). Thus, *A. stephensi* threatens malaria disease reduction and elimination efforts in Africa that have successfully lowered malaria transmission rates due in part to urbanization and improved housing in the past decade (Hay et al., 2005; Mathanga et al., 2016). This concern of an invasive urban malaria vector in the HoA led the WHO to recommend increased vector surveillance, the use of larvicides and the removal or modification of breeding sites in urban and peri-urban environments to mitigate and prevent the further spread of *A. stephensi* in Africa (WHO, 2019). Population models predict that uncontrolled range expansion of *A. stephensi* could lead to a substantial increase in at-risk populations (Sinka et al., 2020), including a potential 50% increase of malaria cases in Ethiopia (Hamlet et al., 2022). In fact, a recent malaria outbreak in Dire Dawa City in eastern Ethiopia in the spring of 2022 (Tadesse et al., 2023; preprint) exemplifies the threat of more malaria outbreaks associated with *A. stephensi* range expansions. Thus, the invasion of *A. stephensi* leading to a resurgence of malaria in the HoA could lead to the same trend further into Africa's interior countries.

An understanding of the history of introduction(s), establishment(s) and potential of these incoming *A. stephensi* sources in the HoA is needed not only to predict future expansions but also to establish where they may be effectively controlled. Specifically, establishing population structure of the invading *A. stephensi* populations is essential for uncovering vector invasion routes, distribution dynamics and evolution in its new environments. However, to demarcate source(s) and the demographic history of founding population(s), it is essential to not only sample at the leading edge of the invasion but also include long-established *A. stephensi* populations (Carter et al., 2018). Specifically, we expect high genetic similarity and a single population model if there is ongoing genetic connectivity (i.e. gene flow) among populations (Hague & Routman, 2016; Lerner, 2001). Alternatively, we expect genetic distinctiveness and an isolation-by-distance model under an invasion scenario followed by sequential founder events (Hague & Routman, 2016; Lerner, 2001). Moreover, if meta-population dynamics exist, then we expect the source of each invasion to harbour the highest levels of genetic diversity as they carry all the diversity present in the subsequent founder populations (Schmidt et al., 2021; Sherpa et al., 2019). Furthermore, understanding how invading populations respond to their new environments is critical to determine whether there may be any geographical limitations to their invasion (Lambrechts et al., 2006), which is currently lacking for *A. stephensi.* For example, genetic diversity found in *Aedes aegypti*, a vector found to share larval habitat with *A. stephensi* (Balkew et al., 2020), showed local adaptation and structured to local environmental conditions (Bennett et al., 2021).

Towards reconstructing *A. stephensi* invasion, establishment and expansion, we take a landscape genomics approach by assessing thousands of nuclear loci and mitochondrial DNA for *A. stephensi* ranging across eastern Ethiopia and Somaliland. In addition to characterizing population structure and genetic connectivity, we associate standing genetic diversity with various environmental factors to understand how these may be contributing to their expansion. Together, these data will not only help us understand how *A. stephensi* invaded the region but also inform targeted vector control approaches and surveillance of this invasive malaria species in the HoA.

## 2 ∣ METHODS

### 2.1 ∣ Sample descriptions

**FIGURE 1:**
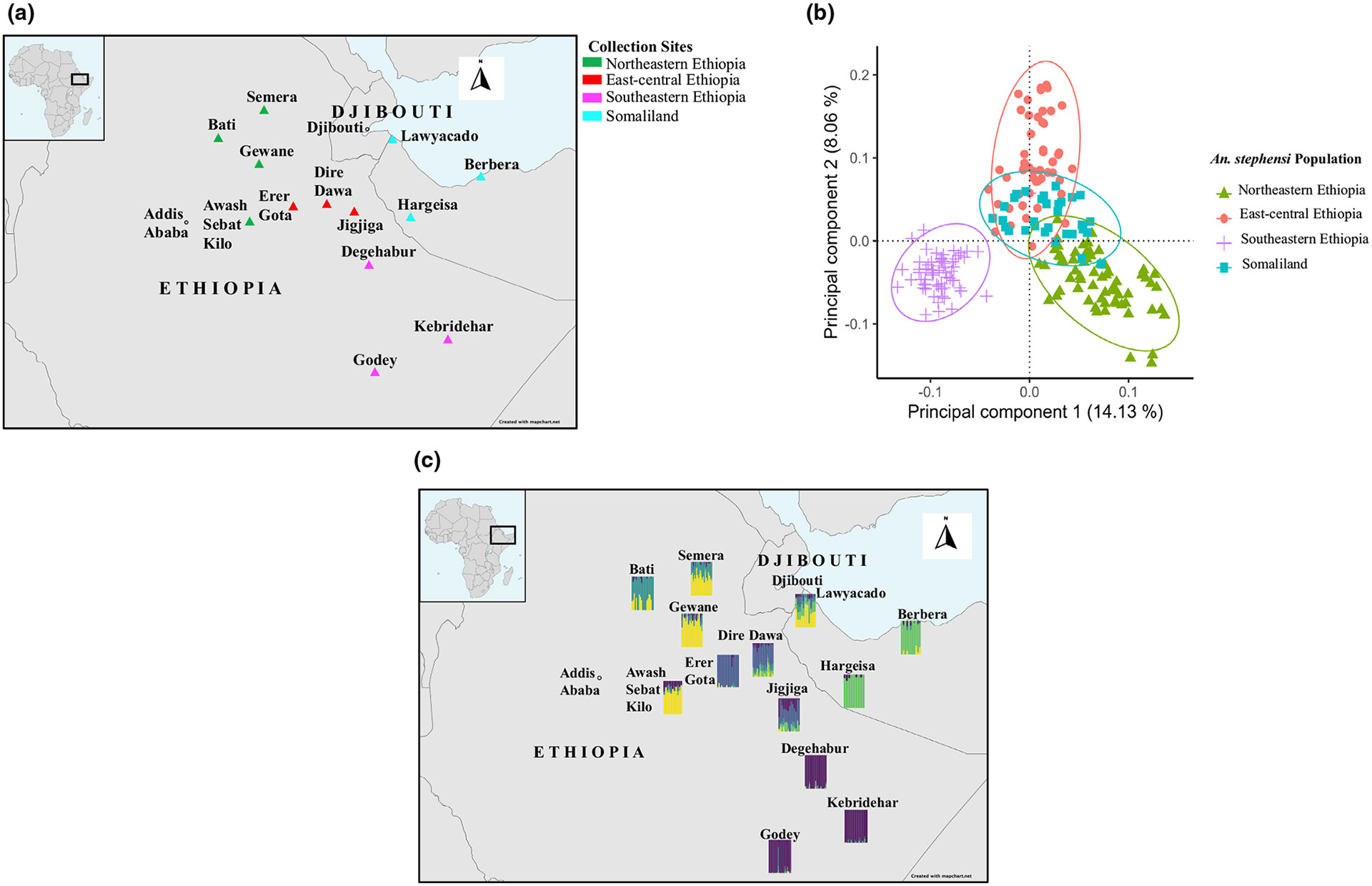
(a) *Anopheles stephensi* sampling locations in eastern Ethiopia and Somaliland. Green triangles represent populations from northeastern Ethiopia (Bati, Gewane, Semera and Awash Sebat Kilo), red triangles represent populations from east-central Ethiopia (Dire Dawa, Erer Gota and Jigjiga), purple triangles represent populations from southeastern Ethiopia (Degehabur, Godey and Kebridehar), and populations from Somaliland (Hargeisa, Berbera and Lawyacado) are colour-coded in blue. (b) Scatterplot of subgroup variation based on principal component analysis (PCA). The amounts of variation explained by each principal component (PC 1 on the *x*-axis, PC 2 on the *y*-axis) are given in percentages. *A. stephensi* population subgroups are colour-coded as described in panel (a). (c) Map with collection site-specific admixture plots.

Samples originated from 13 locations where *A. stephensi* was found across eastern Ethiopia and Somaliland and collected in 2018 and 2020, respectively (Table 1; also see Ali et al., 2022 and Balkew et al., 2020). Briefly, adult mosquitoes were collected using pyrethrum spray sheet collections (PSC) and Centers for Disease Control (CDC) light traps. Larvae and pupae of *Anopheles* were collected from different larval breeding habitats per site, including artificial water containers, to reduce sampling siblings and reared to adults in field insectaries, and then morphologically and molecularly identified as *A. stephensi*. We further categorized collection sites in eastern Ethiopia (northeastern, east-central and southeastern) based on proximity to specific major roads and relative location across sites. ‘Northeastern’ sites included the northern-most sites (Bati, Gewane, Semera and Awash Sebat Kilo) off the B11 and A1 roads (i.e. the road from Mille town to Kombolcha town and the main road between Addis Ababa and Djibouti, respectively). ‘East-central’ sites (Dire Dawa, Erer Gota and Jigjiga) were located in the centre relative to the rest of the collection sites along the A10 road (i.e. the road between Addis Ababa and Degehabur) running west/east. ‘South-eastern’ sites were the southern-most sites (Degehabur, Godey and Kebridehar) off the A10 road running north/south (Table 1, Figure 1a; also see Carter et al., 2021). Being 21.6 km away, we used Lawyacado in this nuclear dataset as a proxy for Djibouti City, where *A. stephensi* was first detected in the HoA.

### 2.2 ∣ DNA extraction

Genomic DNA was extracted from a total of 214 (173 lab-reared and 41 wild-caught) adult *A. stephensi* mosquitoes using Qiagen DNeasy Blood and Tissue kit (Qiagen). DNA quality was assessed on a 1% agarose gel to ensure high-molecular-weight bands and quantified using Nanodrop One Spectrophotometer (Thermo Fisher Scientific Inc.) to ensure a minimum concentration of 20 ng/μL.

### 2.3 ∣ ddRAD-seq library preparation, sequencing and bioinformatics

Genome-wide single nucleotide polymorphism (SNP) data were collected using the double-digest restriction-site-associated DNA sequencing (ddRAD-seq) protocol outlined in Lavretsky et al. (2015), but with fragment size selection following Hernández et al. (2021). Briefly, genomic DNA was enzymatically fragmented using SbfI and EcoRI restriction enzymes, and Illumina TruSeq compatible barcodes were ligated to the sticky ends generated for demultiplexing purposes. The libraries were quantified and pooled in equimolar concentrations, and the multiplexed library was sequenced on Illumina HiSeq X using single-end 150 bp chemistry at Novogene (Novogene CO., Ltd.; see detailed methods, Document S1). Raw Illumina reads have been deposited in NCBI's Sequence Read Archive (Accession # SAMN31227709–SAMN31227956).

Raw Illumina reads were first demultiplexed based on perfect barcode/index sequences using the script *ddRADparser.py* (DaCosta & Sorenson, 2014). We then used Trimmomatic (Bolger et al., 2014) to trim or discard poor-quality sequences using a PHRED score of ≥30 to ensure only high-quality sequences were retained. We then used the Burrows–Wheeler Aligner (bwa; Li & Durbin, 2011) to align the remaining quality reads to the *A. stephensi* reference genome (Accession PRJNA661063; Chakraborty et al. (2022)). Next, samples were sorted and indexed in Samtools (Li et al., 2009). Samples were then genotyped using the ‘mpileup’ function in BCFtools (Li, 2011). At the BCFtools mpileup genotyping step, we filtered out any base pair with PHRED score < 25 and sequences with average PHRED score < 30. Lavretsky et al. (2020) described these methods in detail. In short, bioinformatics followed Lavretsky et al. (2020) with all steps through genotyping automated using custom in-house Python scripts (Python scripts available at https://github.com/jonmohl/PopGen; Lavretsky et al., 2020).

### 2.4 ∣ Nuclear population structure

Nuclear population structure was assessed across samples using an independent set of biallelic SNPs filtered in PLINK v.1.9 (Purcell et al., 2007) for singletons (i.e. minimum allele frequency (--maf 0.004) and any SNP missing ≥20% of data across samples (--geno 0.2)). Independence between SNPs was based on pairwise analysis of linkage disequilibrium (LD) (--indep-pairwise 2 1 0.5) in which one of two linked SNPs are randomly excluded if an LD correlation factor (*r*^2^) > .5 is obtained.

First, we identified variation among samples with a principal component analysis (PCA) using the --pca function in PLINK v.1.9 (Purcell et al., 2007) and visualized with the R package ggplot2 (Wickham, 2016). Next, individual maximum likelihood population assignment probabilities were attained across samples using ADMIXTURE v.1.3 (Alexander et al., 2009), and datasets were formatted using PLINK v.1.9 (Purcell et al., 2007). Each ADMIXTURE analysis was run with a 10-fold cross-validation (CV) and with a quasi-Newton algorithm to accelerate convergence (Zhou et al., 2011). To limit possible stochastic effects, each analysis was based on 1000 bootstraps for each population *K* value of 1–10. The block relaxation algorithm for point estimation was used for each analysis and terminated once the log-likelihood of the point estimation increased by <0.0001. The optimum population value was based on the average of CV errors across the analyses per *K* value. ADMIXTURE assignment probability outputs were visualized using the R package ggplot2 (Wickham, 2016). Additionally, we evaluated patterns of co-ancestry using fineRADstructure (Malinsky et al., 2018), which infers a matrix of co-ancestry coefficient based on the distribution of identical or nearest neighbour haplotypes among samples. Co-ancestry at each locus is equally divided among all individuals with identical haplotypes, or in the case of a unique allele with the nearest neighbour haplotype (Malinsky et al., 2018). Hence, rare haplotypes characterized by rare SNPs, which are on average of more recent origin (Kimura & Ohta, 1973), contribute the most to the co-ancestry index, providing a measure that highlights recent co-ancestry. A burn-in of 100,000 iterations, followed by 100,000 Markov chain Monte Carlo iterations, was completed, followed by tree building using default parameters. To visualize the results, we used the R scripts fineradstructureplot.r and finestructurelibrary.r (available at http://cichlid.gurdon.cam.ac.uk/fineRADstructure.html).

Additionally, nucleotide diversity (*π*), counts of segregating SNPs (S), Tajima's *D* and pairwise estimates of fixation index (*F*_st_) by site were calculated in the R package PopGenome (Pfeifer et al., 2014) and pairwise *F*_st_ by SNP locus was calculated with VCFtools (Danecek et al., 2011). Finally, we used the Mantel test statistic (*r*) implemented in the R package Adegenet to test for any isolation-by-distance effects by assessing the correlation between genetic and geographic distances (Jombart, 2008).

### 2.5 ∣ Genetic network

To uncover genetic connectivity among the sampled populations, we performed a network analysis using EDENetworks (Kivelä et al., 2015), which allows network analyses based on genetic distance matrices without a prior assumption. The network consists of nodes representing populations connected by edges/links weighted by their *F*_st_-based Reynolds' genetic distances (*D*; Reynolds et al., 1983), which provide the strength of connectivity between pairs of populations (Kivelä et al., 2015). The thicker the edge/link, the stronger the genetic connectivity between the two populations. Moreover, node size is proportional to the cumulative weighted edge linkages for each population. Thus, the larger the node the higher the connectivity hub or sink. Statistical confidence of the nodes was evaluated using 1000 bootstrap replicates. Nodes that appear in the top 5 and top 1 lists of betweenness centrality (BC) values (number of shortest genetic paths passing through a node) can be considered as statistically significant (Kivelä et al., 2014).

### 2.6 ∣ Updated mitochondrial DNA genetic diversity and structure

*Anopheles stephensi* cytochrome oxidase subunit 1 (COI) sequences were retrieved from previously published data from eastern Ethiopia (*n* = 191, Carter et al. (2021)), Somaliland (*n* = 33, Ali et al. (2022)) and Djibouti (*n* = 20, de Santi et al. (2021)). Population genetic statistics were generated in DNAsp version 5 (Rozas et al., 2003) for each site from the studies listed above. The statistics generated included the number of polymorphic (segregating) sites (s), number of haplotypes (*h*), haplotype diversity (*H*_d_), nucleotide diversity (*π*) and average number of nucleotide differences (*k*). Population differentiation between sites was determined through pairwise *F*_st_ (differentiation based on haplotype frequencies only) in Arlequin version 3.5.2.2 software (Excoffier & Lischer, 2010). To test the significance of the derived pairwise *F*_st_, 100 permutations were performed, and *p*-values generated. Significance was set at alpha < 0.001. Next, we mapped mitochondrial COI haplotype proportions across sampled regions. Haplotype numbering is the same as in Carter et al. (2021). *Anopheles stephensi* COI sequence alignment was created in MAFFT version 7 (Katoh & Standley, 2013). Finally, a COI phylogeographic tree was re-constructed using a maximum likelihood approach through RAxML (Randomized Accelerated Maximum Likelihood, Stamatakis (2014)) following methods described in Carter et al. (2021). Trees were annotated in FigTree (http://tree.bio.ed.ac.uk/software/figtree/).

### 2.7 ∣ Genotype–environment association

To better understand factors contributing to *A. stephensi* genetic variation and expansion across eastern Ethiopia and Somaliland, we performed a genotype–environment association test following the gradient forest (GF) modelling approach detailed in Brown et al. (2022). Briefly, we used a total of 29 environmental predictor variables, which are reported to influence the ecology and expansion of *A. stephensi* across eastern Ethiopia (Balkew et al., 2020; Sinka et al., 2020) and Somaliland (Ali et al., 2022; Sinka et al., 2020). Specifically, our environmental climate variables include 19 Bioclimatic variables from the CHELSA v2.1 database (Karger et al., 2017, 2021), seasonal and annual vegetation indices (NDVI, EVI), elevation (STRM), land cover (Sentinel 2), net primary productivity (NPP) from the USGS AppEEARS database (https://lpdaacsvc.cr.usgs.gov/ appeears) and population density from NASA Socioeconomic Data and Applications Center (SEDAC, https://sedac.ciesin.columbia.edu; Table S4). In order to differentiate the effects of annual versus seasonal vegetation processes, we calculated an average annual, first rainy season (April) and second rainy season (October) value in the HoA for NDVI and EVI based on data collected from 2000 to 2019.

Following the approach of Brown et al. (2022); also see Bay et al. (2018) and Fitzpatrick and Keller (2015), we used a machine learning regression tree-based analysis (i.e. random forests from the R package GradientForest (Ellis et al., 2012)). Briefly, GF detects the effects of environmental predictor variables across the landscape on turnover in allele frequencies between sample sites. For use as response variables, we used the R package PopGenome (Pfeifer et al., 2014) to calculate the minor allele frequencies from the previously filtered independent biallelic autosomal SNPs. Additionally, we filtered any SNP that was polymorphic in fewer than five sample sites in order to reduce the potential for false positives. Using a large number of trees (*N* = 5000), GF produced an *R*^2^ ranked list of weighted importance for all environmental and human disturbance variables. To assess the strength of actual GF models, we randomized the environmental predictor data in relation to sampling sites; we then compared the performance of these 100 randomized models to our observed data. To visualize the GF model across eastern Ethiopia and Somaliland, we extracted values for the top five predictor variables from random points generated across this range. We then used a principal component analysis (PCA) to summarize the transformed values from the top five predictor variables (based on GF goodness of fit *R*^2^ weighted importance) for each point. Based on the GF goodness of fit *R*^2^, only SNPs with positive *R*^2^ values are significantly associated with genetic turnover associated with environmental predictor variables (Ellis et al., 2012; Fitzpatrick & Keller, 2015). Finally, we transformed the top three principal components to create a RGB colour scale that was used to visualize patterns of adaptive genetic diversity across the landscape. In the end, colours reflect associations between allele frequencies and the environmental predictor variables that allow us to draw conclusions about how the environment has affected genetic diversity and putatively driven adaptation.

## 3 ∣ RESULTS

### 3.1 ∣ Nuclear population structure

Across samples, we recovered 2070 ddRAD-seq autosomal loci (5675 base pairs) that met our criteria for sequencing coverage and missing data. Mean coverage per sample was 135× (range 10–179×). Moreover, genetic diversity and population structure analyses were based on 1680 (of 2070) independent biallelic SNP dataset collected across samples.

**FIGURE 2:**
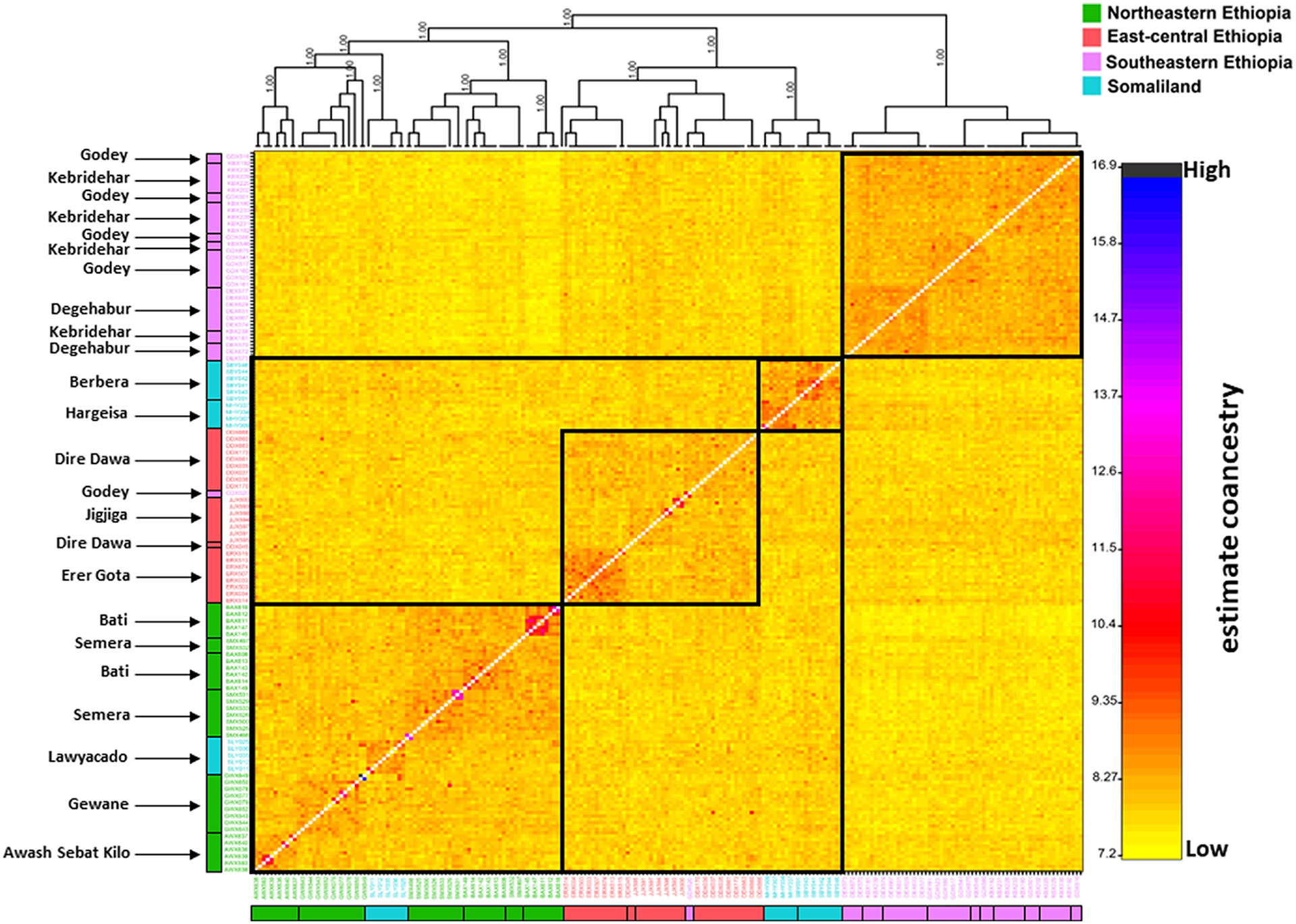
Co-ancestry matrix based on fineRADstructure analysis of autosomal ddRAD-seq loci of *Anopheles stephensi*. The dendrogram depicts clustering of individual samples based on the pairwise matrix of co-ancestry coefficients. Pairwise coefficients of co-ancestry are colour-coded from low (yellow) to high (blue) according to the scale shown on the right. Samples are colour-coded based on sample descriptions (see Table 1, Figure 1). Square black box demarcations identify individuals showing higher co-ancestry.

**FIGURE 3:**
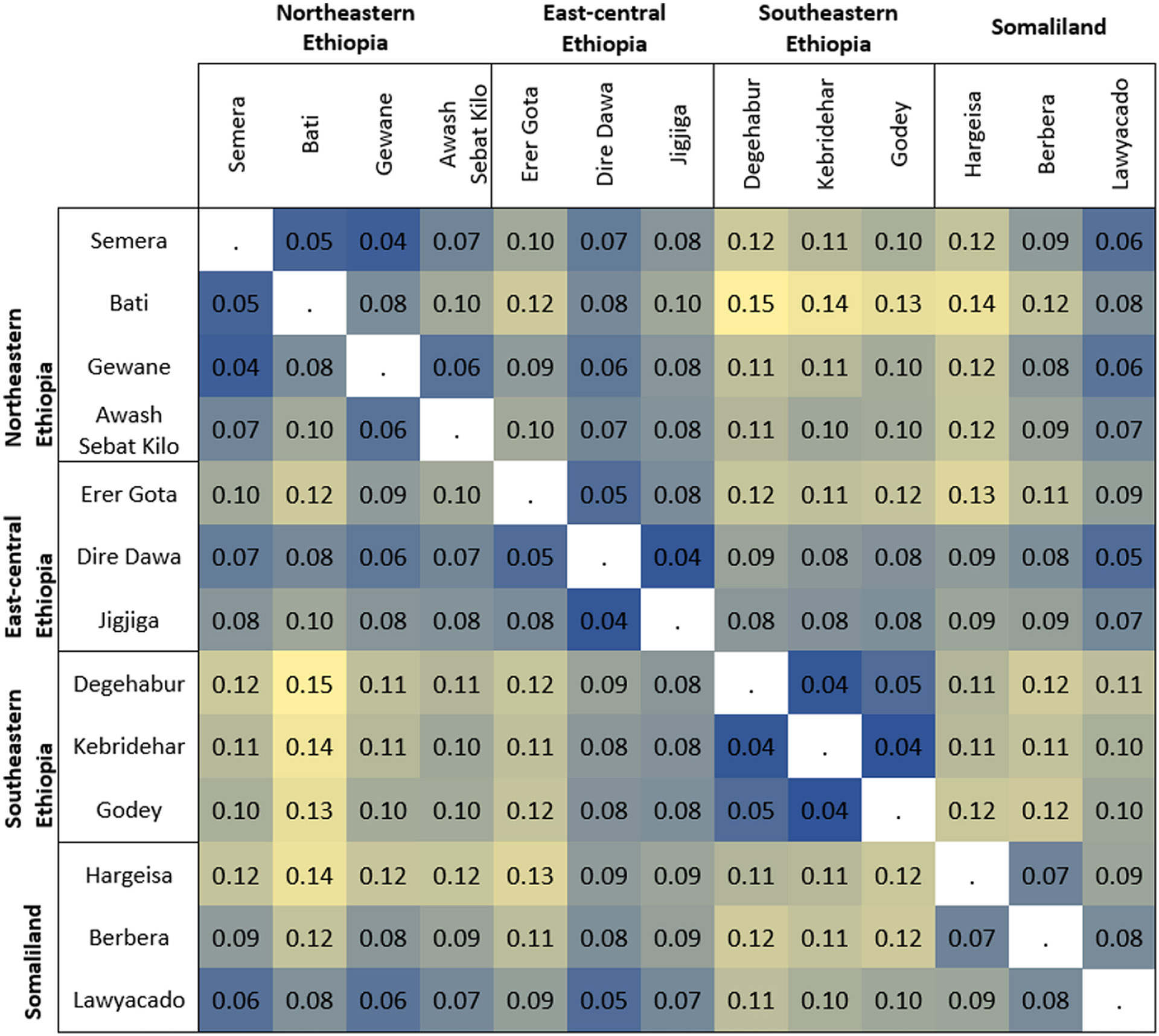
Population pairwise *F*_st_ based on autosomal loci. Colour gradient based on *F*_st_ values (yellow = highest, dark blue = lowest).

In the *A. stephensi* populations that were sampled, the lowest genetic diversity was observed in the southeastern Ethiopia region and the highest in the northeastern Ethiopia region, as assessed by basic summary statistics (Table 2). The lowest nucleotide diversity was observed in Godey (*π* = 0.1942), and the highest nucleotide diversity was observed in Awash Sebat Kilo (*π* = 0.2233) (Table 2). *Anopheles stephensi* populations from the southeastern Ethiopian region were also found to have the highest Tajima's *D* values, 1.37, 1.47 and 1.13 for Degehabur, Kebridehar and Godey, respectively, indicating a lack of rare variants relative to neutral expectation (Table 2; Tajima, 1989). Moreover, Somaliland *A. stephensi* populations had high nucleotide diversity even with limited sample sizes compared with the Ethiopian *A. stephensi* populations (Table 2). Specifically, in the Somaliland region, the lowest nucleotide diversity was observed in Hargeisa (*π* = 0.2014, *N* = 9) and the highest nucleotide diversity was observed in Lawyacado (*π* = 0.2191, *N* = 11; Table 2). Assessing population structure using principal component analysis (PCA; Figure 1b; Figure S1), ADMIXTURE (Figure 1c; Figure S2) and fineRADstructure (Figure 2) analyses consistently recovered four semidiscrete genetic clusters and with general genetic structuring and pairwise *F*_st_ estimates (Figure 3) consistent with an isolation-by-distance (IBD) pattern (Mantel test observation: *r* = .54, *p*-value < .001; Figure S3). On average, 91 out of 1680 SNPs were recovered as outliers driving this observed IBD pattern based on the *F*_st_ values per SNP locus (Figure S4, Table S1). In general, plotting the first two principal components clustered samples regionally (Figure 1b; Figure S1), while ADMIXTURE analysis based on an optimum population *K* model of 5 more specifically identified four main genetic similarities across the regions that include (1) Lawyacado, Semera, Bati, Gewane and Awash Sebat Kilo; (2) Erer Gota, Dire Dawa and Jigjiga; (3) Degehabur, Kebridehar and Godey; and (4) Hargeisa and Berbera (Figure 1c). From these genetic clusters, Lawyacado, Dire Dawa and Jigjiga showed substantially higher admixture proportions (Figure 1c; Figure S2). In fact, the derived co-ancestry matrix from the fineRADstructure analysis (Figure 2) provided further resolution and confirmation of the above genetic clusters. Specifically, fineRADstructure results revealed some levels of recent co-ancestry between Awash Sebat Kilo, Gewane, Lawyacado, Semera and Bati *A. stephensi*, followed by Erer Gota, Dire Dawa and Jigjiga *A. stephensi*, then Hargeisa and Berbera *A. stephensi*, and finally Degehabur, Kebridehar and Godey *A. stephensi* (Figure 2). Similar to PCA and ADMIXTURE analyses, FineRADstructure analysis also showed Degehabur, Kebridehar and Godey *A. stephensi* populations as an isolated branch in the fineRADstructure dendrogram suggesting a separate founder event in the southeastern Ethiopian region (Figure 2).

Pairwise *F*_st_ estimates also revealed genetic differentiation within these studied populations, with *F*_st_ values ranging from 0.04 to 0.15 (Figure 3). Southeastern Ethiopia *A. stephensi* populations were again highly differentiated (Figure 3), whereas the Dire Dawa *A. stephensi* population in east-central Ethiopia was least differentiated from the other studied *A. stephensi* populations (Figure 3).

### 3.2 ∣ Genetic network

**FIGURE 4:**
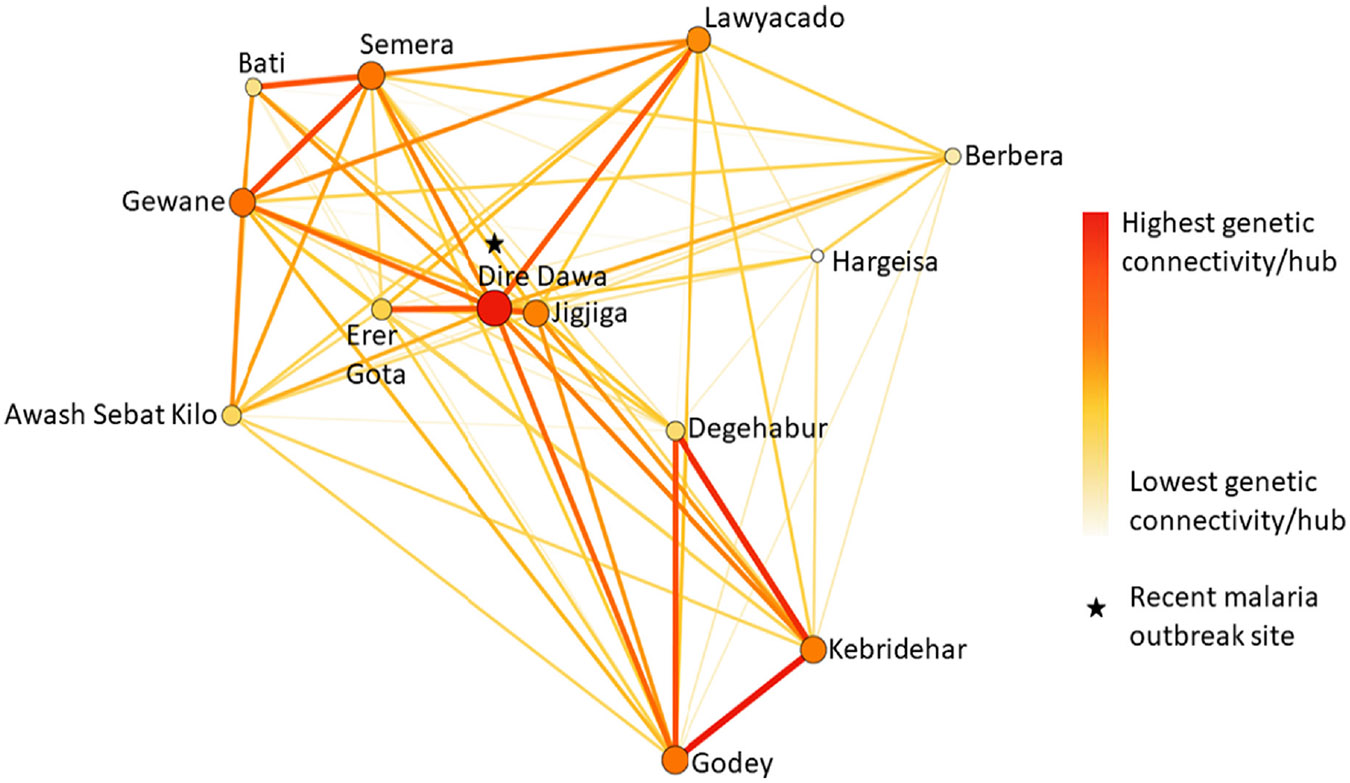
Genetic network of *Anopheles stephensi* populations in eastern Ethiopia and Somaliland. Network nodes represent populations/hubs and links represent weighted genetic distances/genetic connectivities. The figure is produced by EDENetworks based on a genotype autosomal ddRAD-seq loci matrix by applying a single realization of bootstrapping with 0.85 percentage of nodes at each location and thresholded at 0.15. The colours and sizes of the links represent the strength of genetic connectivity from lowest (white) to highest (red). The colours and sizes of the nodes represent the cumulative weighted links from lowest (white) to highest (red). The sample sites are Erer Gota, Dire Dawa, Jigjiga, Berbera, Hargeisa, Lawyacado, Awash Sebat Kilo, Gewane, Semera, Bati, Degehabur, Kebriderar and Godey.

Network reconstruction was based on the same 1680 independent biallelic SNP dataset. Although all sites were recovered to be interconnected, Dire Dawa was recovered as the statistically significant node in the genetic network, followed by Godey, Semera, Jigjiga, Kebridehar, Gewane and Lawyacado (Figure 4), as they appeared in both the top 5 and top 1 lists of nodes from the bootstrapping procedure (Figure S5). The network revealed high genetic connectivity between Dire Dawa and sites in northeastern Ethiopia (Semera (SM), Gewane (GW)), east-central Ethiopia (Erer Gota (ER), Jigjiga (JJ)), southeastern Ethiopia (Kebridehar (KB), Godey (GD)) and Somaliland (Lawyacado (SL); Figure 4). This finding suggests Dire Dawa is a major *A. stephensi* hub among the studied populations. Other pairs of sites with high genetic connectivity were Lawyacado (SL) and Semera (SM), Bati (BA) and Semera (SM), Gewane (GW) and Semera (SM) and between the trio Degehabur (DE), Kebridehar (KB) and Godey (GD; Figure 4).

### 3.3 ∣ Updated mitochondrial DNA genetic diversity and structure

**FIGURE 5:**
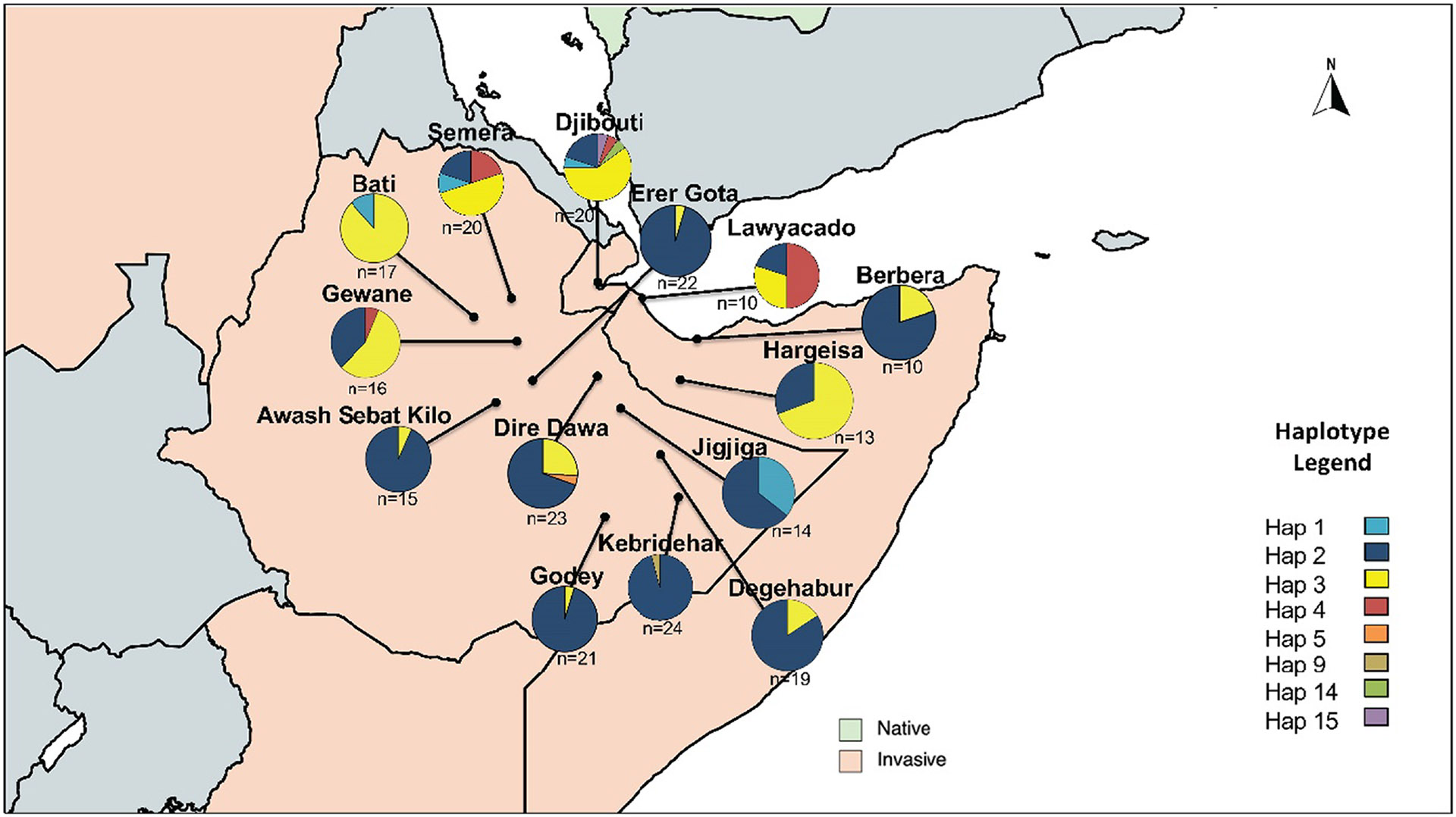
Map of HoA *Anopheles stephensi* COI haplotypes frequencies.

Mitochondrial COI analyses were based on 317 base pairs of over-lapping sequences for 244 samples (Table S2). Within the HoA, we recovered eight COI haplotypes, with different frequencies across the region (Figure 5; Table S2). The haplotypes are based on five polymorphic sites (Table S2). The most common haplotype was Haplotype 2 observed in Djibouti, Somaliland and Ethiopia (Figure 5). The highest haplotype diversity was observed in Lawyacado, Semera and Djibouti (*H*_d_ > 0.620) and the lowest was in Erer Gota, Godey and Kebridehar (*H*_d_ < 0.100) (Table S2). We also calculated pairwise *F*_st_ based on genetic distances to determine the relationship between the major subregions in the HoA. The highest average *F*_st_ values were for Bati (0.582), Kebridehar (0.387) and Lawyacado (0.371), and the lowest average *F*_st_ was for Berbera (0.110), Dire Dawa (0.174) and Degehabur (0.214) (Table S3). A phylogeographic tree was created using *A. maculatus* as an outgroup. Overall, the tree provides low-to-moderate bootstrap (bs) support for the nodes (max bs = 57, excluding outgroup; Figure S6). In the tree, haplotypes 1, 3 and 4, predominately found in northeastern and east-central Ethiopia, Djibouti and Somaliland, all cluster together with moderate-to-low bootstrap support (bs = 57) (Figure S6). Within this cluster, Hap 1 is basal to Hap 3 and Hap 4 and another rarer haplotype found in Djibouti. This cluster is sister to Hap 2, the most prevalent haplotype and the one with the broadest geographic distribution (Figure S6).

### 3.4 ∣ Genotype–environment association

Overall, GF models were not found to be significant compared with randomized models (SNPs with positive *R*^2^ value: *N* = 153; mean *R*^2^ value: *N* = 0.160) (Figure S8), suggesting that genotypic turnover does not seem to be strongly associated with the environment (Figures S7A,B). However, GF still recovered 137 SNPs (out of 1680, 8.2%) with a positive GF goodness of fit *R*^2^ value, representing a number of alleles that are associated with the environment. While three of the top five environmental predictors were related to seasonal changes such as precipitation of coldest quarter (Bio19), temperature seasonality (Bio4) and normalized difference vegetation index (NDVI.April) (Figures S7C and S9), the strongest predictive variable in the region was the mean diurnal range (Bio2; Figures S7C and S9). Finally, while the GF PCA showed a minimal signal of local adaptation (Figure S7B), there is clear genotypic turnover between Somaliland and east-central and southeastern Ethiopia (Figure S7A).

## 4 ∣ DISCUSSION

### 4.1 ∣ *Anopheles stephensi* population structure reveals dispersal patterns within the Horn of Africa

We provide the most complete analysis of the population structure for the recently invaded and currently expanding *A. stephensi* in the HoA. First, our data showed an *A. stephensi* population structure that appears to be consistent with an isolation-by-distance pattern (Figure S3) with various levels of genetic connectivity, including across international borders (Figure 1b; Figure S1). This observed population structure seems to be driven by about 91 outlier SNPs potentially due to Bottlenecks or any kind of selection (Figure S4, Table S1). Additionally, the highest level of nuclear diversity was observed closest to Djibouti, where *A. stephensi* was first detected in the HoA [i.e. Lawyacado (Somaliland) and Semera (Northeastern Ethiopia)] or at transportation hubs [i.e. Awash Sebat Kilo (Northeastern Ethiopia) and Dire Dawa (east-central Ethiopia)] (Figure 1a, Table 2). This pattern of diversity indicates these sites contain older populations. These findings support *A. stephensi* populations dispersal patterns from north central to all the other regions in the HoA, suggesting that north central HoA populations are more likely to be the source populations leading to subsequent founding populations moving outward, with resulting new populations characterized by low genetic variation (Howe et al., 1991). Indeed, southeastern *A. stephensi* populations were differentiated from Somaliland, northeastern and east-central Ethiopia *A. stephensi* populations (Figures 1b,c and 3). This finding coupled with the low genetic diversity observed in these populations (Table 2) suggests a potential recent founder event such as a Bottleneck for the southeastern Ethiopian *A. stephensi* populations compared with other studied populations (Haddrill et al., 2005; Nei et al., 1975). This scenario is further supported by the co-ancestry matrix model, which revealed a separate dendrogram branch for southeastern Ethiopia *A. stephensi* populations, but a shared one for *A. stephensi* populations in Somaliland, northeastern and east-central Ethiopia regions (Figure 2).

Furthermore, the discovery of Dire Dawa as a significant hub (Figure 4; Figure S5) coupled with the isolation-by-distance population structure pattern suggests the potential role of roads and human transportation in the dispersal of *A. stephensi* from one site to another. For instance, Dire Dawa which was reported as the site of a recent malaria outbreak associated with *A. stephensi* (Tadesse et al., 2023) (preprint) had a high genetic connectivity with Lawyacado (SL), which is 21.6 km away from Djibouti City (Figure 4). Thus, this high genetic connectivity observed between Dire Dawa and Lawyacado despite an isolation-by-distance population structure pattern could potentially be explained by the fact that there is a direct train route between Djibouti City and Dire Dawa. Overall, the unbalanced sample size between the Ethiopian and Somaliland studied sites posed a limitation. However, the number of independent biallelic SNPs recovered helped our analyses remain informative. Thus, future studies will need to test the potential impact of roads and human transportation on the dispersal of *A. stephensi* within the HoA.

### 4.2 ∣ Updated *Anopheles stephensi* mitochondrial DNA structure confirms patterns of dispersal into the Horn of Africa

Analysing HoA *A. stephensi* mtDNA COI gene provided further support for the observed nuclear population structure and dispersal patterns of *A. stephensi* into the HoA region. Our findings showed distinct genetic clusters and levels of mtDNA COI genetic variations among the studied *A. stephensi* populations in the HoA region that suggest a radiative dispersal pattern from north central to the other regions (Figure 5; Figure S6). Specifically, Djibouti City *A. stephensi* had the most genetic variation, followed by *A. stephensi* from the closest sites such as Lawyacado and Semera, in Somaliland and Ethiopia, respectively (Figure 5; Table S2). Additionally, the low mitochondrial COI diversity observed in *A. stephensi* populations from the east-central and southeastern Ethiopian regions, coupled with the fact that one or two of the prevalent Djiboutian COI haplotypes were found in every site, again supports potential sequential founder events of *A. stephensi* from Djibouti City outward in the HoA (Figure 5; Figure S6 and Table S2). Again, this mtDNA COI population structure leading to the observed dispersal patterns could potentially be explained by the fact that Ethiopia, a landlocked country, relies on seaports in Djibouti City and Berbera for goods imports and exports. The role that maritime sea transports and port cities play in invasive mosquito species incursion in new geographic areas has been well documented (Ahn et al., 2023; Ammar et al., 2019; Hulme, 2009; Swan et al., 2022). Thus, future work will require the testing of this hypothesis for the movement of the invasive *A. stephensi* populations by a simultaneous and continued sampling of port cities and inland cities across the region to decipher the potential role of seaports on the introduction, dispersal and genetic variation of *A. stephensi* in the HoA.

### 4.3 ∣ Minimal environmental association with *Anopheles stephensi* genetic variation

Our genotype–environment association analysis revealed a minimal environmental association with genetic turnover in eastern Ethiopia and Somaliland (Figures S7 and S8). These results emphasize the more impactful role that genetic drift is currently having on genetic diversity in the HoA, as *A. stephensi* continues to expand. In general, the weak predictive power of GF is likely due to the recent establishment of *A. stephensi*, meaning that selective pressures have not had sufficient time to act throughout the genome in a way that would be detected from our relatively low genome coverage (i.e. ~0.13%). However, other studies have identified rapid changes in *A. stephensi* going from primarily seasonal to year-round activity that could be the result of strong selective forces (Seyfarth et al., 2019; Sinka et al., 2020; Whittaker et al., 2023), such as the annual temperature variables GF identified as high ranking in predictive power (Figures S7C and S9). These kinds of temperature variations were also reported to impact *A. stephensi* activity and survival in lab settings (Paaijmans et al., 2010, 2013). Therefore, while we do not see a strong signal in our study, tracking the signal over time could be beneficial in understanding if local adaptation is occurring. Future work will require continued sampling and full genome analyses to better understand how genetic diversity continues to respond to environmental pressures in the HoA.

### 4.4 ∣ Potential *Anopheles stephensi* expansion controls and future malaria outbreaks

Our findings have significant implications for *A. stephensi* vector control in the HoA. First, the population structure and dispersal patterns could inform strategies to manage the current invasion and prevent the further spread of *A. stephensi* within the HoA.

Also, the finding of Dire Dawa, a recent A. stephensi-associated malaria outbreak site, as a significant hub may aid in predicting/preventing other malaria outbreaks within the region. Therefore, we highly recommend heightening vector control surveillance in the observed significant hub sites (i.e. Dire Dawa, Godey, Semera, Jigjiga, Kebridehar, Gewane and Lawyacado) to prevent potential future malaria outbreaks. Also, a genotype–environment association model with a longitudinal nuclear SNP dataset could help monitor and determine critical environmental and climate variables contributing to establishing this invasive *A. stephensi* population, as ecological variables are known factors influencing the development and vectorial capacity of established insect vectors (Alto et al., 2017; Holmes & Benoit, 2019; Paaijmans et al., 2009; Zapletal et al., 2018). Thus, incorporating both genomic and environmental analyses into future *A. stephensi* vector surveillance could significantly aid in designing timely and targeted vector control strategies against this malaria vector in the HoA and beyond.

## 5 ∣ CONCLUSION AND FUTURE DIRECTIONS

Overall, our present study answered many questions regarding the genetic population structure and dispersal patterns of *A. stephensi* in the HoA. Genetic drift via sequential founder events, rather than adaptation to local environmental conditions, seems to be the main evolutionary force currently impacting *A. stephensi* genetic variation in the HoA though we posit that natural selectivity processes will begin to take shape as this mosquito further establishes in the region. Our study also provides a tool to predict and prevent potential malaria outbreaks in a timely manner by identifying important *A. stephensi* hubs in the region. However, future studies could benefit from longitudinal and more extensive *A. stephensi* sampling across the HoA. Finally, with our genomic data being the only African *A. stephensi* SNPs dataset, we recommend the incorporation of genome-wide multilocus data into future studies of African *A. stephensi* for higher resolution comparative analyses between populations and better prediction of *A. stephensi* dispersal within the region and beyond.

## Supplementary Material

Supplemental 1

Supplemental 2

## Figures and Tables

**TABLE 1:** *Anopheles stephensi* sample description from 13 populations. Altitude in metres above sea level (masl).

Population ID	Site	Samples passing QC	Altitude (masl)	Coordinates
Northeastern Ethiopia	Northeastern Ethiopia			
BA	Bati	21	2055	11°1′92″ N, 40°0′7″ E
GW	Gewane	16	617	10°1′66″ N, 40°6′46″ E
SM	Semera	21	431	11°7′94″ N, 41°0′08″ E
East-central Ethiopia	East-central Ethiopia			
AW	Awash Sebat Kilo	11	916	8.9′89″ N, 40°1′64″ E
DD	Dire Dawa	20	1178	9°5′96″ N, 41°8′54″ E
ER	Erer Gota	17	1090	9°5′56″ N, 41°3′84″ E
JJ	Jigjiga	14	1657	9°3′51″ N, 42°7′93″ E
Southeastern Ethiopia	Southeastern Ethiopia			
DE	Degehabur	21	1065	8°2′23″ N, 43°5′58″ E
GD	Godey	20	294	5°9′49″ N, 43°5′53″ E
KB	Kebridehar	22	532	6°7′38″ N, 44°2′77″ E
Somaliland	Somaliland			
SL	Lawyacado	11	7	11°27′30″ N, 43°15′47″ E
SB	Berbera	11	10	10°26′17″ N, 45°1′12″ E
MH	Hargeisa	9	1334	9°34′6″ N, 44°4′55″ E

**TABLE 2:** Basic summary statistics based on autosomal loci.

Collection site	Sample size *N*	Nucleotide diversity π	Counts of segregating SNPs S	Tajima's *D*
Northeastern Ethiopia				
Semera	21	0.2205	1332	0.74
Bati	21	0.2036	1174	0.95
Gewane	16	0.2163	1238	0.71
Awash Sebat Kilo	11	0.2233	1167	0.71
East-central Ethiopia				
Erer Gota	17	0.2137	1171	0.94
Dire Dawa	20	0.2199	1327	0.64
Jigjiga	14	0.2057	1155	0.65
Southeastern Ethiopia				
Degehabur	21	0.1958	1036	1.37
Kebridehar	22	0.2028	1057	1.47
Godey	20	0.1942	1062	1.13
Somaliland				
Hargeisa	9	0.2014	977	0.82
Berbera	11	0.2071	1078	0.73
Lawyacado	11	0.2191	1234	0.74

## Data Availability

DNA sequences: Bioproject PRJNA888109 and Genbank accessions SAMN31227709–SAMN31227956.
