# Supplementary material for: Population genomic analyses reveal population structure and major hubs of invasive Anopheles stephensi in the Horn of Africa
Source: Mol Ecol. Author manuscript; Available in PMC 2026 Jan 21. (PMC12822364; doi:10.1111/mec.17136)
Supplement: Supplemental 1 [file NIHMS2042731-supplement-Supplemental_1.pdf]

Title: Population genomic analyses reveal population structure and major hubs of invasive *Anopheles stephensi* in the Horn of Africa

Jeanne N. Samake[1], Philip Lavretsky[2], Isuru Gunarathna[1], Madison Follis[1], Joshua I. Brown[3], Said Ali[4], Solomon Yared[5], Tamar E. Carter[1*]

1. Department of Biology, Baylor University, Waco, Texas, USA
2. Department of Biological Sciences, University of Texas at El Paso, El Paso, Texas, USA
3. Department of Life, Earth, and Environmental, West Texas A&M University, Texas, USA
4. Ministry of Health Somaliland, Hargeisa, Somalia
5. Department of Biology, Jigjiga University, Jigjiga, Ethiopia

Tamar Carter: tamar_carter@baylor.edu *corresponding author

Supplemental information

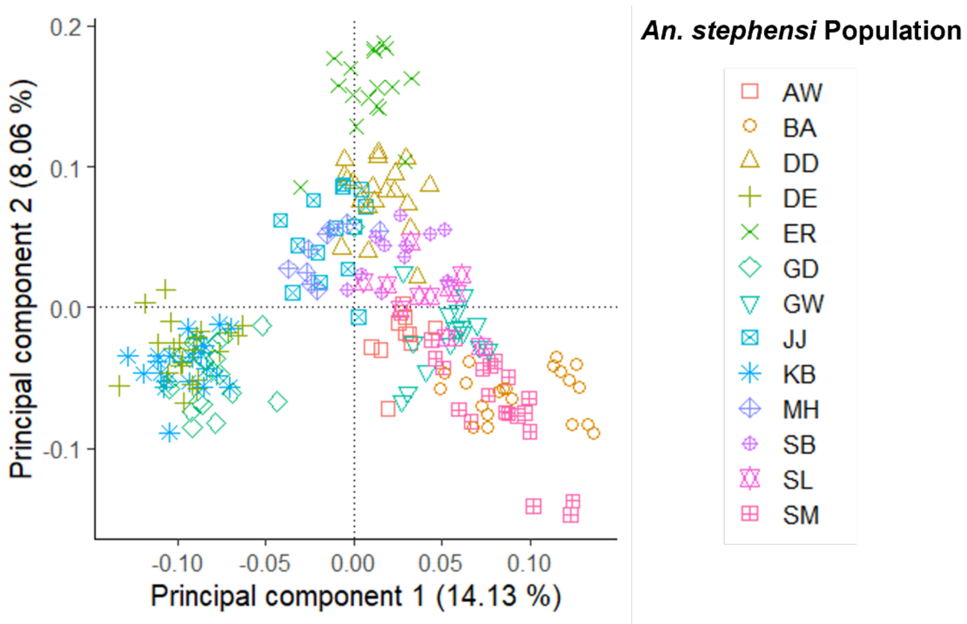

Figure S1. Principal component analysis (PCA) of individual *An. stephensi* population in eastern Ethiopia and Somaliland. The amounts of variation explained by each principal component (PC 1 on the x-axis, PC 2 on the y-axis) are given in percentages. The sample sites are Erer Gota (ER), Dire Dawa (DD), Jigjiga (JJ), Berbera (SB), Hargeisa (MH), Lawyacado (SL), Awash Sebat Kilo (AW), Gewane (GW), Semera (SM), Bati (BA), Degehabur (DE), Godey, (GD), and Kebriderar (KB).

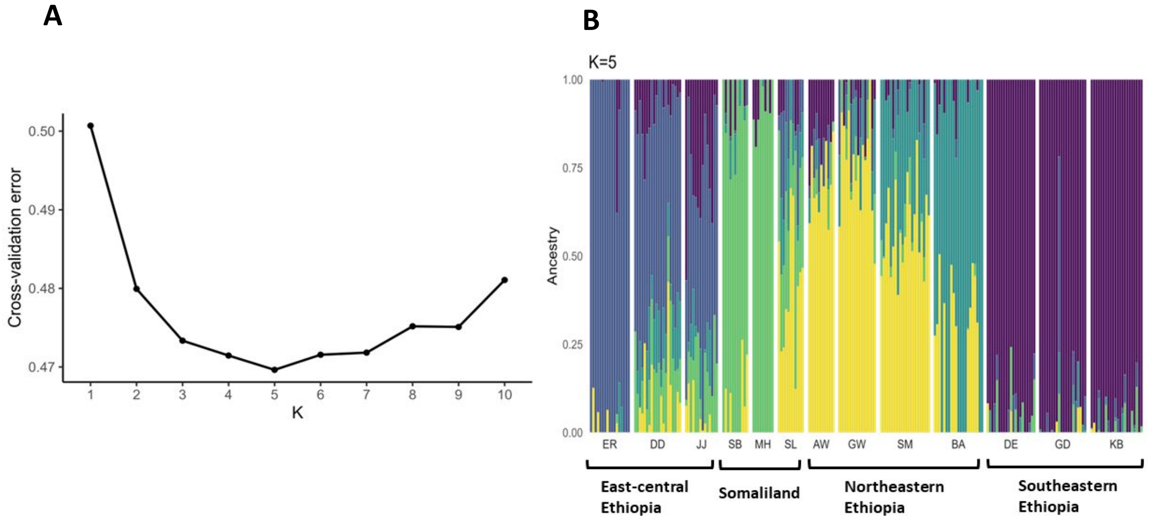

Figure S2. A) Cross-validation errors of K values **B)** Ancestry plot based on ADMIXTURE analysis. Each barplot represent an individual. The height of each colored bar represents the probability of assignment to the corresponding ancestry, when assuming the presence of five ancestral populations (K = 5). The sample sites are Erer Gota (ER), Dire Dawa (DD), Jigjiga (JJ), Berbera (SB), Hargeisa (MH), Lawyacado (SL), Awash Sebat Kilo (AW), Gewane (GW), Semera (SM), Bati (BA), Degehabur (DE), Godey, (GD), and Kebriderar (KB).

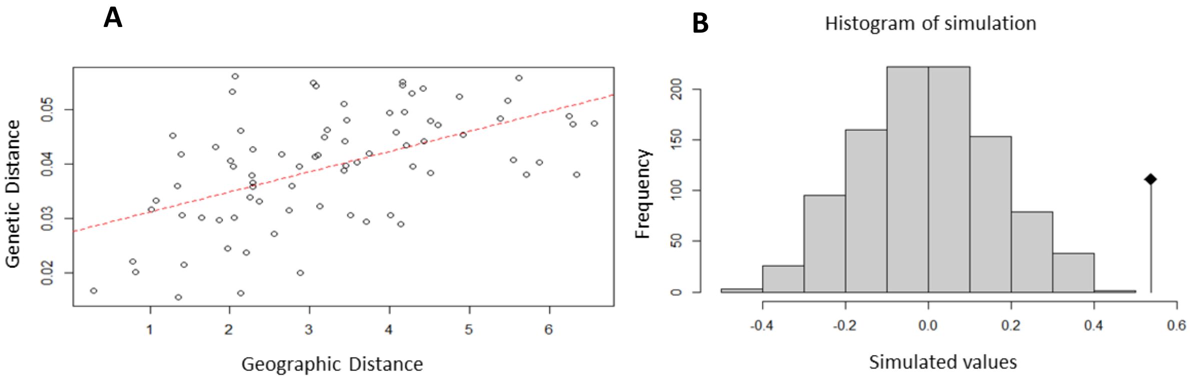

Figure S3. A) Scatterplot of isolation-by-distance showing the log of geographic distance by the log of genetic distance based on 1680 biallelic SNPs and **(B)** Mantel Test histogram of simulation results based on 999 replicates (Mantel test observation: 0.54, p-value < 0.001). The original value of the correlation between the distance matrices is represented by the dot, while histograms represent permuted values in the absence of spatial structure. Significant spatial structure would therefore result in the original value being outside of the reference distribution.

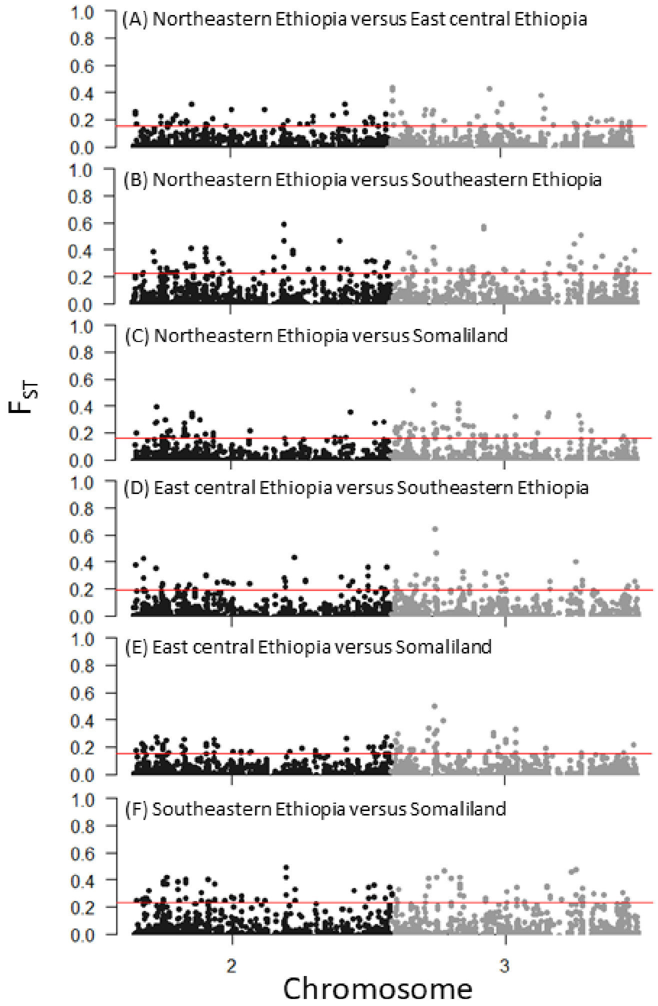

Figure S4. *Anopheles stephensi* SNPs F_{st} values per chromosome per studied regions. Red line represents the 95th percentile demarcation. SNPs F_{st} values above the red line are considered significant.

Table S1. *Anopheles stephensi* SNPs count with significant F_{st} values.

**Regions**	**SNPs count**
Northeastern Ethiopia vs. East central Ethiopia	85
Northeastern Ethiopia vs. Southeastern Ethiopia	91
Northeastern Ethiopia vs. Somaliland	93
East central Ethiopia vs. Southeastern Ethiopia	84
East central Ethiopia vs. Somaliland	93
Southeastern Ethiopia vs. Somaliland	98
**Average**	91

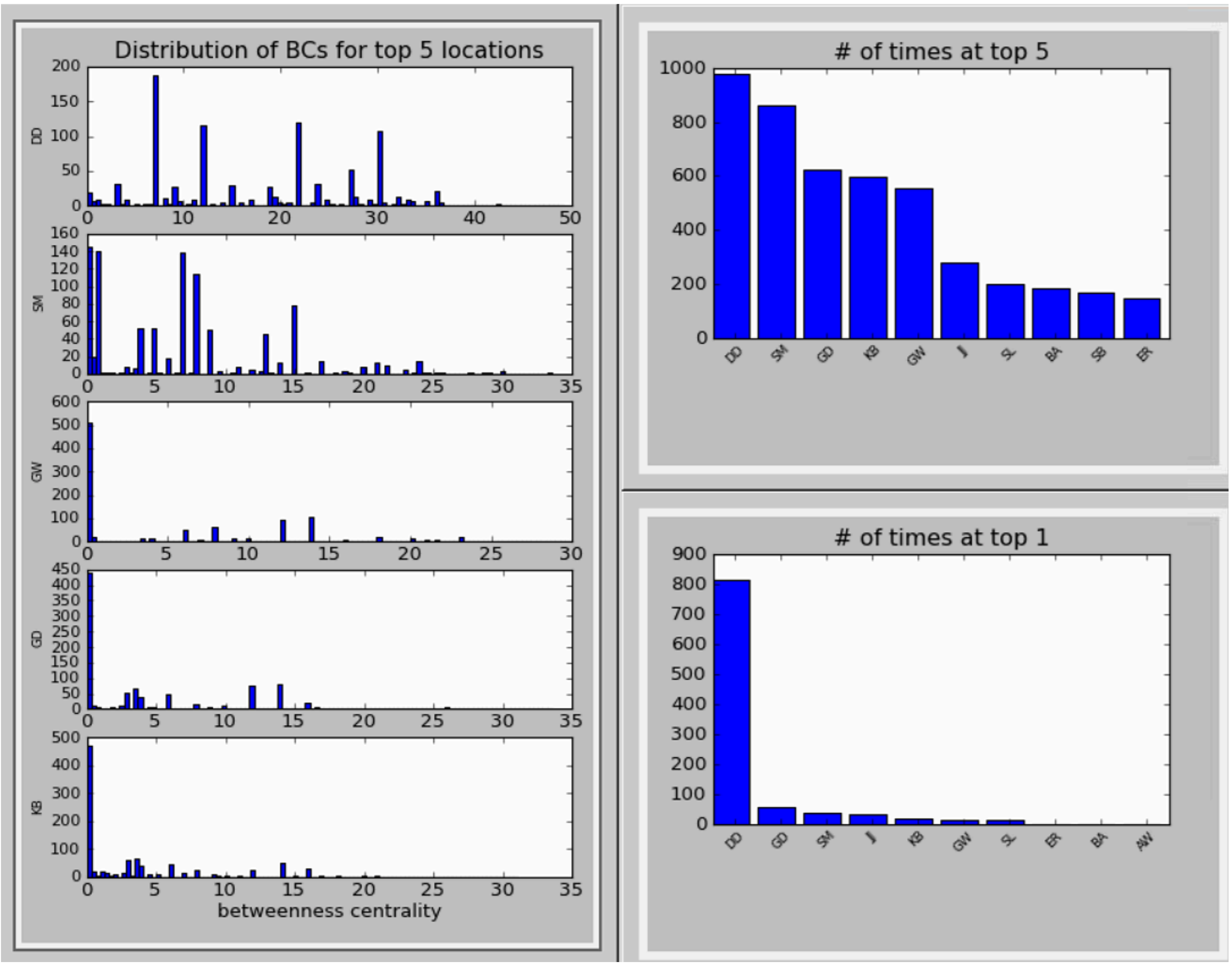

Figure S5. Bootstrapping results for betweenness centrality with number of repetitions (1000) and thresholded at 0.85 for eastern Ethiopia and Somaliland *An. stephensi* genetic networks.

Table S2. Basic summary statistics based on mtDNA COI loci.

Collection site	Sample size N	Number of polymorphic (segregating) sites, S	Number of haplotypes, h	Haplotype diversity, Hd	Nucleotide diversity π	Average number of nucleotide differences, k
**Northeastern Ethiopia**						
Semera	20	3	4	0.695	0.00379	1.2
Bati	17	1	2	0.221	0.0007	0.221
Gewane	16	3	3	0.575	0.00363	1.15
Awash Sebat Kilo	15	2	2	0.133	0.00084	0.267
**East-central Ethiopia**						
Erer Gota	22	2	2	0.091	0.00057	0.182
Dire Dawa	23	3	3	0.466	0.00282	0.893
Jigjiga	14	1	2	0.495	0.00156	0.495
**Southeastern Ethiopia**						
Degehabur	19	2	2	0.281	0.00177	0.561
Kebridehar	24	1	2	0.083	0.00026	0.083
Godey	21	2	2	0.095	0.0006	0.19
**Somaliland**						
Hargeisa	13	2	2	0.462	0.00291	0.923
Berbera	10	2	2	0.356	0.00224	0.711
Lawyacado	10	3	3	0.689	0.00435	1.378
**Djibouti**						
Djibouti City	20	4	6	0.621	0.00349	1.105

Table S3. Average F_{st} based on COI genetic distances.

	**Average F_{st}**
**Berbera**	0.110
**Dire Dawa**	0.174
**Degehabur**	0.214
**Gewane**	0.244
**Awash**	0.266
**Semera**	0.275
**Jigjiga**	0.276
**Djibouti**	0.306
**Godey**	0.308
**Erer Gota**	0.314
**Hargeisa**	0.370
**Lawyacado**	0.371
**Kebridehar**	0.387
**Bati**	0.582

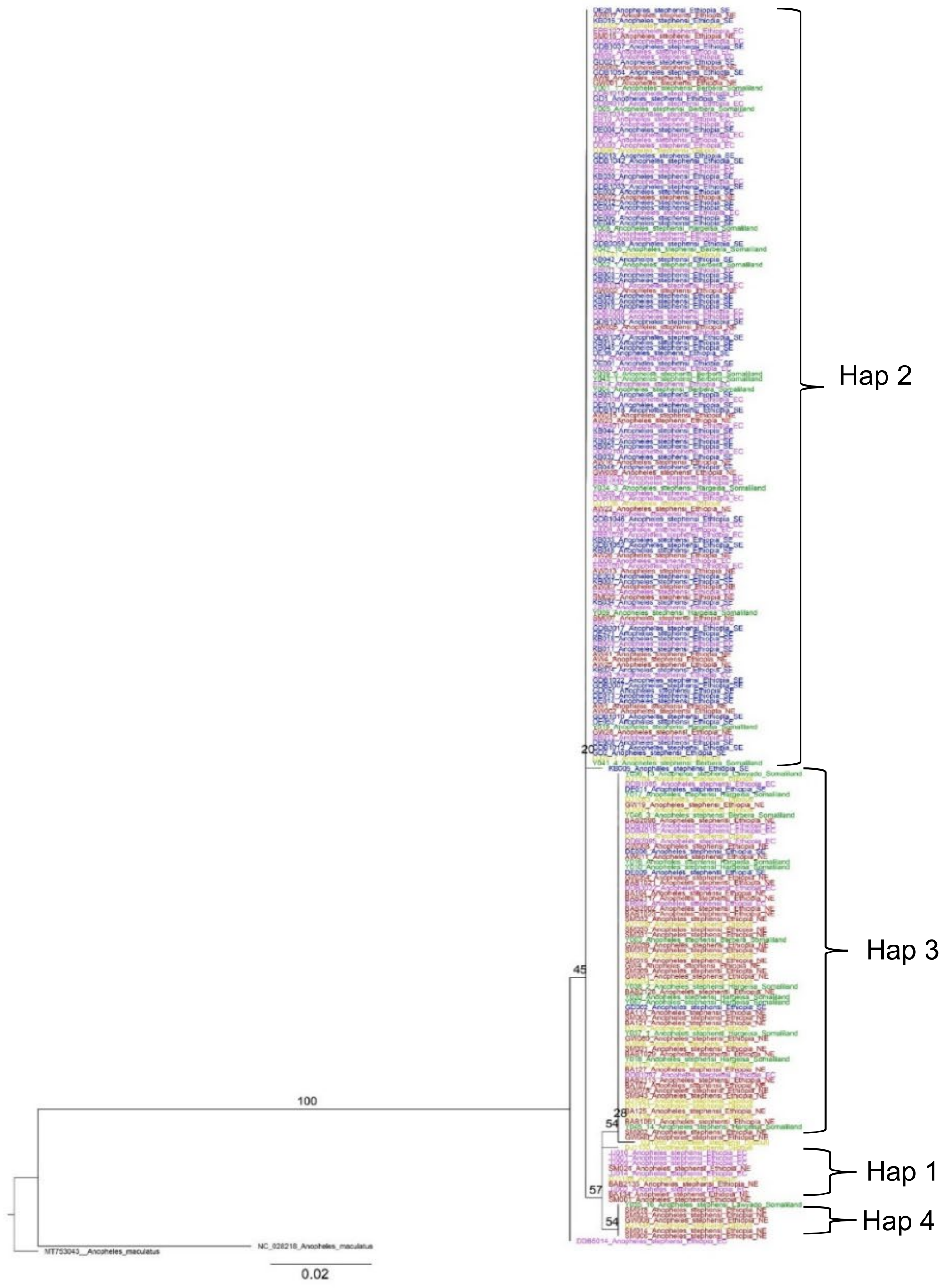

Figure S6. Phylogenetic analysis of HoA *An. stephensi* cytochrome oxidase subunit I (COI) sequenced data colored by subregion as follows: red = northeastern Ethiopia, pink = east central Ethiopia, blue = southeastern Ethiopia, green = Somaliland, and yellow = Djibouti.

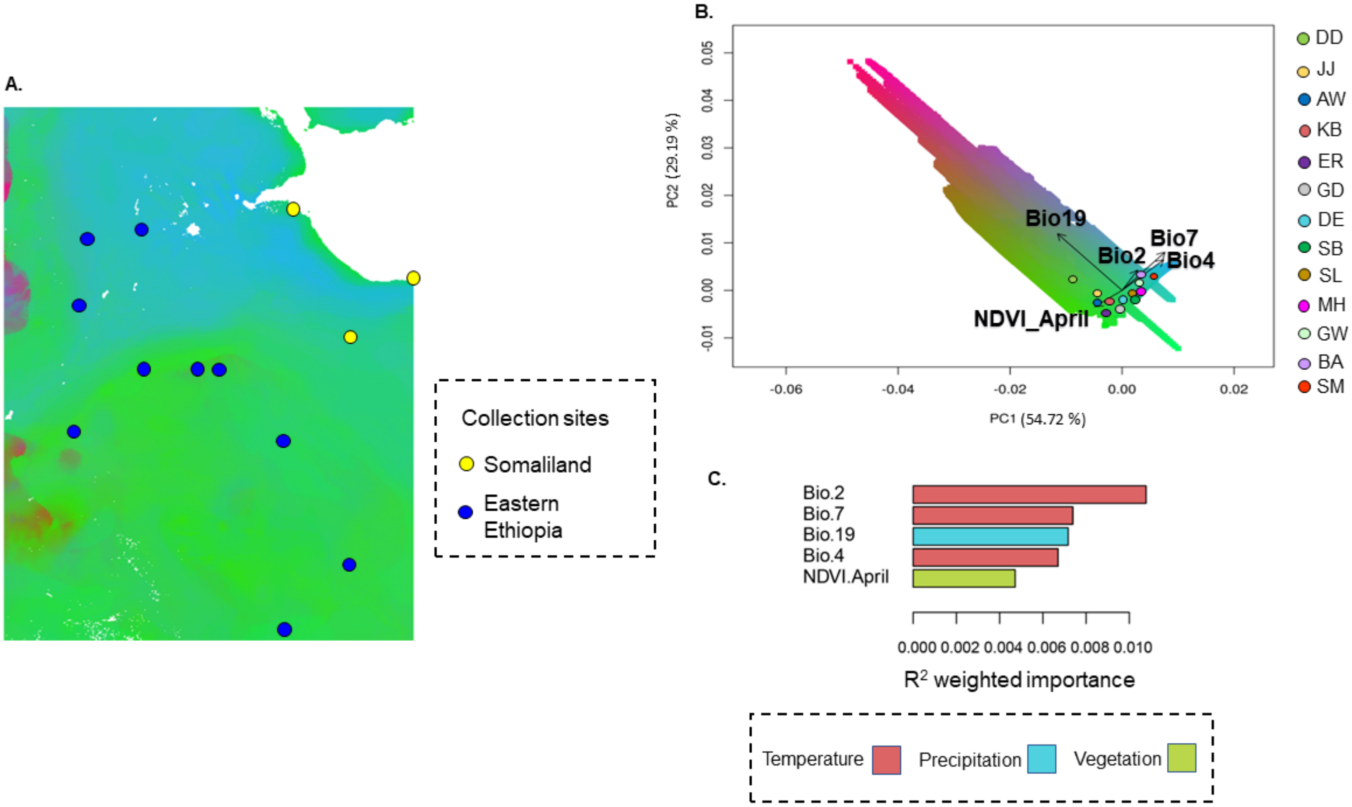

Figure S7. A) Genotype-environment association model from gradientForest (GF), where **(B)** the PCA **(C)** of the top five most predictive environmental variables, mean diurnal range (Bio2), temperature annual range (Bio7), precipitation of coldest quarter (Bio19), temperature seasonality (Bio4), and normalized difference vegetation index (NDVI.April), based on R^2 weighted importance are mapped across eastern Ethiopia and Somaliland for *An. stephensi*. In general changes in color reflect changes in allele frequency in relation to the environment. Sample sites are Somaliland (Berbera (SB), Lawyacado (SL), Hargeisa (MH)) and Eastern Ethiopia (Semera (SM), Bati (BA), Gewane (GW), Awash Sebat Kilo (AW), Erer Gota (ER), Dire Dawa (DD), Jigjiga (JJ), Degehabur (DE), Kebridehar (KB), Godey (GD)).

Table S4. List of environmental variables used for genotype-environment association testing in GradientForest.

Variable	Description	Unit
BIO1	Mean annual temperature	°C
BIO2	Mean diurnal range	°C
BIO3	Isothermality	°C
BIO4	Temperature seasonality	°C
BIO5	Max temperature of warmest month	°C
BIO6	Min temperature of coldest month	°C
BIO7	Temperature annual range	°C
BIO8	Mean temperature of wettest quarter	°C
BIO9	Mean temperature of driest quarter	°C
BIO10	Mean temperature of warmest quarter	°C
BIO11	Mean temperature of coldest quarter	°C
BIO12	Annual precipitation	mm
BIO13	Precipitation of wettest month	mm
BIO14	Precipitation of driest month	mm
BIO15	Precipitation seasonality	mm
BIO16	Precipitation of wettest quarter	mm
BIO17	Precipitation of driest quarter	mm
BIO18	Precipitation of warmest quarter	mm
BIO19	Precipitation of coldest quarter	mm
NDVI_ANNUAL	Normalized Difference Vegetation Index Annual; MOD13A3	
NDVI_APRIL	Normalized Difference Vegetation Index April; MOD13A3	
NDVI_OCTOBER	Normalized Difference Vegetation Index October; MOD13A3	
EVI_ANNUAL	Enhanced Vegetation Index Annual; MOD13A3	
EVI_APRIL	Enhanced Vegetation Index April; MOD13A3	
EVI_OCTOBER	Enhanced Vegetation Index October; MOD13A3	
NPP_ANNUAL	Net Primary Productivity Annual; MOD17A2H	
SRTM	Shuttle radar topography mission; Elevation	m
LAND_COVER	Sentinel-2	m
POPULATION_DENSITY	Gridded Population of the World, Version 4, 2020	

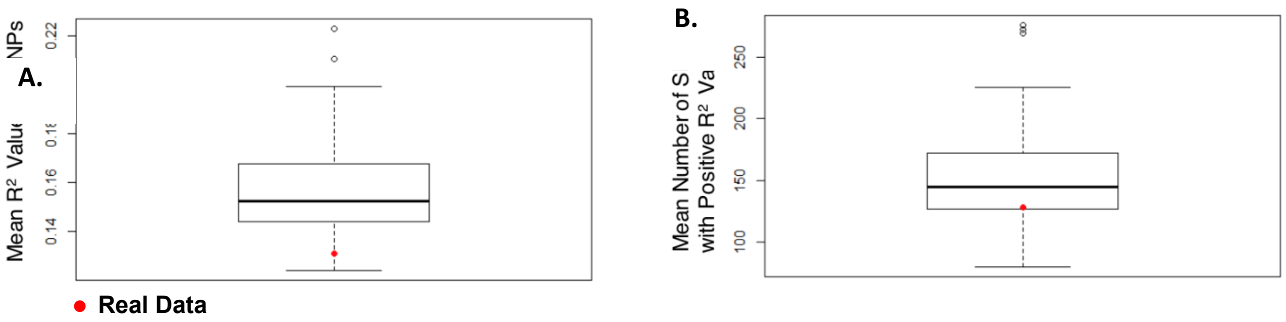

Figure S8. Boxplots of R^2 values from randomized dataset tested in GradientForest compared to the actual data. **A)** Mean of R^2 values of SNPs. **B)** Mean number of SNPs with positive R^2 values.

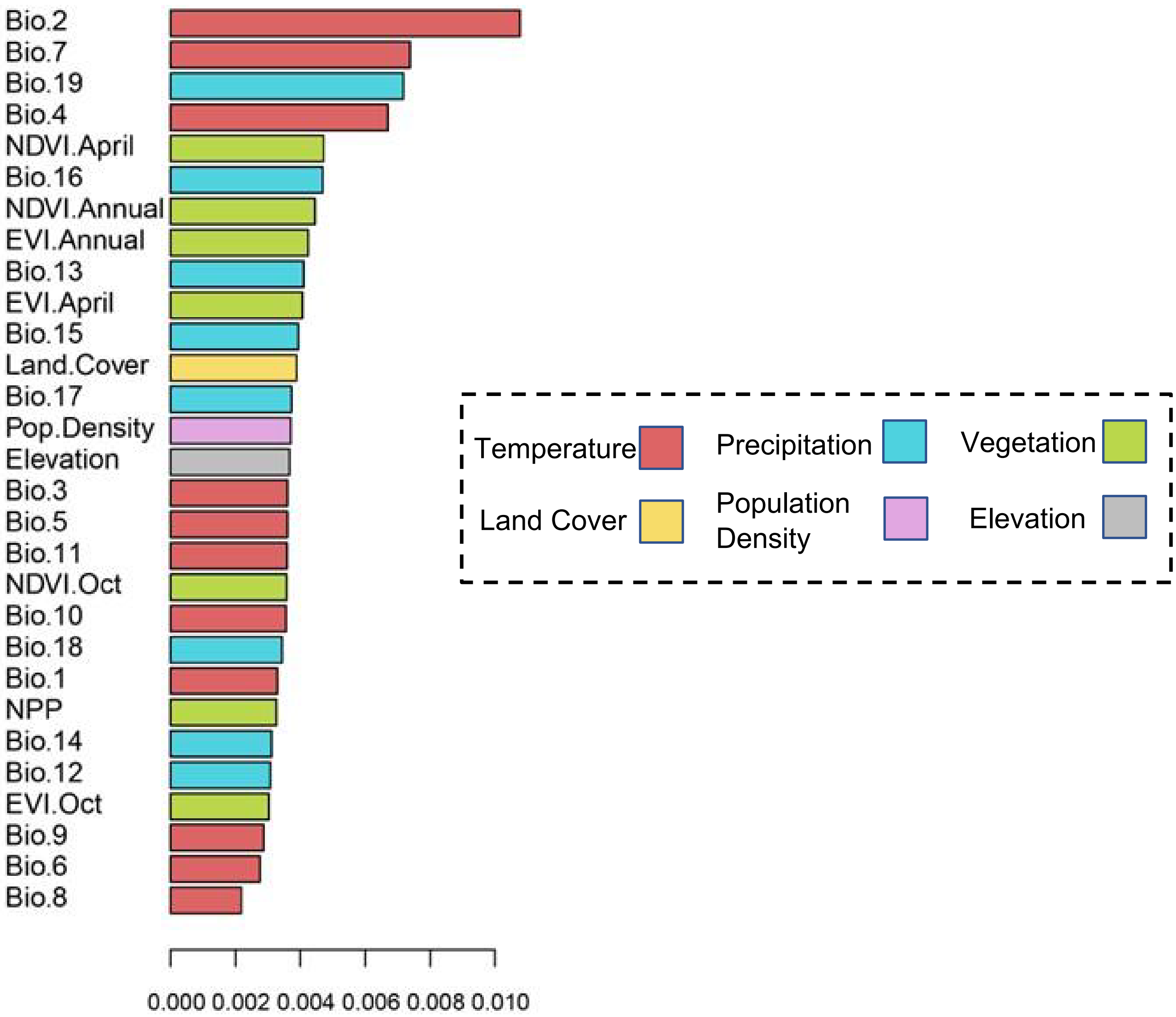

Figure S9. Cumulative R^2 weighted importance ranking of 29 environmental predictor variables from GradientForest.
