# Supplementary material for: Population genomic analyses reveal population structure and major hubs of invasive Anopheles stephensi in the Horn of Africa
Source: Mol Ecol. Author manuscript; Available in PMC 2026 Jan 21. (PMC12822364; doi:10.1111/mec.17136)
Supplement: Supplemental 2 [file NIHMS2042731-supplement-Supplemental_2.pdf]

Supplementary Document S1

Title: Population genomic analyses reveal population structure and major hubs of invasive *Anopheles stephensi* in the Horn of Africa

Jeanne N. Samake[1], Philip Lavretsky[2], Isuru Gunarathna[1], Madison Follis[1], Joshua I. Brown[3], Said Ali[4], Solomon Yared[5], Tamar E. Carter[1*]

1. Department of Biology, Baylor University, Waco, Texas, USA
2. Department of Biological Sciences, University of Texas at El Paso, El Paso, Texas, USA
3. Department of Life, Earth, and Environmental, West Texas A&M University, Texas, USA
4. Ministry of Health Somaliland, Hargeisa, Somalia
5. Department of Biology, Jigjiga University, Jigjiga, Ethiopia

Tamar Carter: tamar_carter@baylor.edu *corresponding author

ddRAD-seq Library Preparation Methods

Genome-wide single nucleotide polymorphism (SNP) data was collected using the double-digest restriction-site associated DNA sequencing (ddRADseq) protocol outlined in Lavretsky et al. (2015), but with fragment size selection following Hernandez et al. (2021). A total of 250 samples with ~20 ng of genomic DNA each were enzymatically fragmented using 1 µL each of SbfI and EcoRI restriction enzymes. Illumina TruSeq compatible adapters and 6 base-pair barcodes were ligated to allow for future de-multiplexing. The adapter-ligated DNA fragments were then size selected using a double-sided size selection based on a total of 0.8x solution of sparQ PureMag beads (Quantabio, MA, USA). First, a right-sided selection for large fragments was completed by adding 0.55x concentration of the total starting ligated DNA solution. The solution was incubated at room temperature for 10 minutes, then transferred to a magnetic plate to rest for 5 minutes or until the mixture became clear. The supernatant containing both target-sized and small-sided DNA fragments was then transferred to new tubes and the beads were discarded. Next, a left-sided size selection against small DNA fragments (<100bp) was completed by adding 0.25x concentration of the total starting ligated DNA solution. The solution was again incubated at room temperature for 10 minutes, then transferred to a magnetic plate to rest for 5 minutes at room temperature or until the mixture became clear. The supernatant containing small-sided DNA fragments (<100bp) was then discarded and the beads were washed twice with 70% ethanol. The beads were then air-dried at room temperature for 2-5 minutes. DNA was re-suspended with 22 µL molecular grade water and eluted for 1 hour, then transferred to a magnetic plate to rest for 1 minute at room temperature or until the mixture became clear. The eluant was then transferred to new tubes. Target size selected DNA fragments were then PCR amplified with Phusion High-Fidelity DNA polymerase, and 10x concentration of forward and reverse RAD primers (Dacosta & Sorenson, 2014) under the following PCR conditions: an initial 30 sec cycle at 98 ^oC, followed by 22 cycles of 10 sec at 98 ^oC, 30 sec at 60 ^oC, and 40 sec at 72 ^oC, with a final extension at 72 ^oC for 5 min. Amplicons were then cleaned using a 1.8x solution of sparQ PureMag beads (Quantabio, MA,

USA) and two 70% ethanol washed before final elution in 40 µL molecular grade water. Library concentrations were quantified using Qubit dsDNA BR Assay Kit (ThermoFisher Scientific, MA, USA) following manufacturer protocols. Samples were then pooled in equimolar amounts and the multiplexed library was sequenced on Illumina Hiseq X using single-end 150 bp chemistry with Novogene (Novogene Inc., CA, USA).

REFERENCES

DaCosta, J. M., & Sorenson, M. D. (2014). Amplification biases and consistent recovery of loci in a double-digest RAD-seq protocol. *PLoS ONE*, *9*(9), e106713. doi:10.1371/journal.pone.0106713

Hernández, F., Brown, J. I., Kaminski, M., Harvey, M. G., & Lavretsky, P. (2021). Genomic Evidence for Rare Hybridization and Large Demographic Changes in the Evolutionary Histories of Four North American Dove Species. Animals : an open access journal from MDPI, 11(9), 2677. https://doi.org/10.3390/ani11092677

Lavretsky, P., Dacosta, J. M., Hernández-Baños, B. E., Engilis, A., Sorenson, M. D., & Peters, J. L. (2015). Speciation genomics and a role for the Z chromosome in the early stages of divergence between Mexican ducks and mallards. *Molecular Ecology*, *24*(21), 5364–5378. doi:10.1111/mec.13402
